# Optimization of Extraction Conditions for Water-Soluble Polysaccharides from the Roots of *Adenophora tetraphylla* (Thunb.) Fisch. and Its Effects on Glucose Consumption on HepG2 Cells

**DOI:** 10.3390/molecules29133049

**Published:** 2024-06-27

**Authors:** Junkai Wu, Xiaohang Zhou, Huifeng Sun, Dan Yu

**Affiliations:** 1 School of Pharmacy, Quanzhou Medical College, Quanzhou 362011, China; wujk1@hotmail.com; 2 Pharmaceutical College, Heilongjiang University of Chinese Medicine, Harbin 150040, China; xiaohang20210101@163.com (X.Z.); 13936243669@139.com (H.S.)

**Keywords:** polysaccharides, Adenophorae Radix, response surface methodology, Box–Behnken design, glucose consumption

## Abstract

The root of *Adenophora tetraphylla* (Thunb.) Fisch. is a common Chinese materia medica and the polysaccharides which have been isolated from the plant are important active components for medicinal purposes. The objective of the current study was to optimize the extraction parameters and evaluate the glucose consumption activity for Adenophorae root polysaccharides (ARPs). The optimization of ARP extraction was evaluated with preliminary experiments and using response surface methodology (RSM). The conditions investigated were 35–45 °C extraction temperature, 20–30 (*v*/*w*) water-to-solid ratio, and 3–5 h extraction time. The antidiabetic effects of ARPs for the glucose consumption activity were evaluated in HepG2 cells. The statistical analyses of the experiments indicated that temperature, water-to-solid ratio, and extraction time significantly affected ARP yield (*p* < 0.01). The correlation analysis revealed that the experimental data were well-aligned with a quadratic polynomial model, as evidenced by the mathematical regression model’s fit. The optimal conditions for maximum ARP yield were 45 °C extraction temperature and 28.47:1 (mL/g) water-to-solid ratio with a 4.60 h extraction time. Extracts from these conditions showed significant activity of promoting cell proliferation from 11.26% (*p* < 0.001) to 32.47% (*p* < 0.001) at a dose of 50 μg/mL to 800 μg/mL and increasing glucose consumption to 75.86% (*p* < 0.001) at 250 μg/mL on HepG2 cells. This study provides a sustainable alternative for the industry since it allowed simplified handling and a specific quantity of ARPs. Furthermore, ARPs might directly stimulate the glucose consumption in the liver and showed no cytotoxicity; therefore, ARPs probably could be taken as a potential natural source of antidiabetic materials.

## 1. Introduction

Polysaccharides are a class of highly bioactive macromolecules, and in recent years, much attention has been paid to their biological activities due to the efficient anti-tumor activity and biosecurity. An increasing number of polysaccharides have been extracted from plants, and they have shown a wide range of positive abilities in medicine and health-care foods, like antitumor, antioxidant, anti-fatigue, anti-inflammatory, anti–anxiety, and anti-aging effects [1,2,3]. Moreover, many plant polysaccharides have shown good antidiabetic effects and may be explored as a new potential natural resource of antidiabetic materials [4,5,6]. Therefore, a growing number of researchers are focusing on the discovery and evaluation of new polysaccharides.

Adenophorae Radix (also known as “Nan shashen” in China), is derived from the root of *Adenophora tetraphylla* (Thunb.) Fisch. or *Adenophora stricta* Miq. in the Chinese Pharmacopoeia [7]. *A. tetraphylla* (Thunb.) Fisch., also known as *Adenophora triphylla*, is a medicinal and edible plant within the Campanulaceae family. It is commonly referred to by various names, including four-leaf Adenophorae Radix, bell grass, tiger’s beard, and rolling pin. It grows mainly in the northeastern, north and eastern areas of China, in Korea, Japan, and the far east of Russia. The roots of *A. tetraphylla* have long been used for the treatment of dry cough attributed to lung dryness, chronic cough arising from yin deficiency, cough accompanied by viscous phlegm, as well as conditions characterized by qi (a vital life force that is believed to flow through all living things) and yin (a fundamental concept that represents the cold, slow, passive, and still aspects of the universe) deficiency. In addition, the tender leaves, stems, and roots from the plant can be used as food due to their rich nutrient contents, such as amino acids, vitamins, phospholipids, etc. [8].

Adenophorae root contains a variety of important chemical components, mainly polysaccharides, alkaloids, coumarins, sitosterols, and essential oil [9]. Radix Adenophorae polysaccharides were obtained through a purification process involving ADS-7 macroporous adsorption resin for decolorization, followed by anion-exchange chromatography using DEAE-52 cellulose and gel filtration on Sephacryl S-300HR. The resulting polysaccharides exhibited a high purity of 98.3% and a molecular weight of 1.8 × 10^4^ Da [10]. The polysaccharide fraction from Adenophorae Radix was composed of a variety of monosaccharides and uronic acids, including xylose, arabinose, glucose, rhamnose, mannose, galactose, glucuronic acid, and galacturonic acid. Notably, arabinose, galacturonic acid, galactose, and glucose were present in comparatively higher quantities. The extraction methods of polysaccharides included hot water extraction, enzymatic hydrolysis, microwave-assisted extraction, ultrasonic wave extraction, etc. [11,12]. The crude polysaccharides are usually obtained by water soaking, reflux extraction, alcohol precipitation, deproteinization, and secondary alcohol precipitation, in that order. Hot water extraction of plant polysaccharides has been widely used owing to low cost and high extraction yield. Compared with a lot of research on the phytochemical analysis of triterpenoids and phenolic glycosides in *A. tetraphylla* [13,14], studies on the extraction and separation of polysaccharides have not gained wide attention until now. A study employed the central composite design to optimize the extraction of water-soluble polysaccharides from Adenophorae Radix; however, the origin of the Chinese materia medica utilized in that research remained unspecified [15]. In the meantime, polysaccharides from Adenophorae Radix (ARPs) are known to possess anti-aging, anti-radiation, anti-obesity, and hypocholesterolemic effects, but *A. tetraphylla* was not the origin of the polysaccharides in most of the studies [9]. There is no information about the potential bioactivity of the ARPs to date.

In the course of hot water extraction, the content of polysaccharides could be affected by some parameters; the extraction temperature, extraction time, and solvent-to-solid ratio are important factors for high yield of polysaccharides. Box–Behnken design (BBD), the most common design for response surface methodology (RSM), has been widely employed in optimizing the effect of independent variables and their interaction on response variables [16]. In terms of these advantages, BBD is widely used to optimize the extraction of bioactive components in Chinese materia medica, such as polysaccharides [17,18], flavonoids [19], alkaloids [20], and steroids [21]. Therefore, the content of ARPs was set as the response value while the extraction temperature, extraction time and number, and ratio of water to solid were considered as the optimization parameters in this study first. Then, the BBD of RSM, followed by canonical and ridge analyses, was applied to optimize the extraction process parameters of ARPs. Finally, the potential effect on their glucose consumption activity in HepG2 was also evaluated.

## 2. Results and Discussion

### 2.1. Effect of Single-Factor on the Extraction Efficiency of ARPs

**Figure 1:**
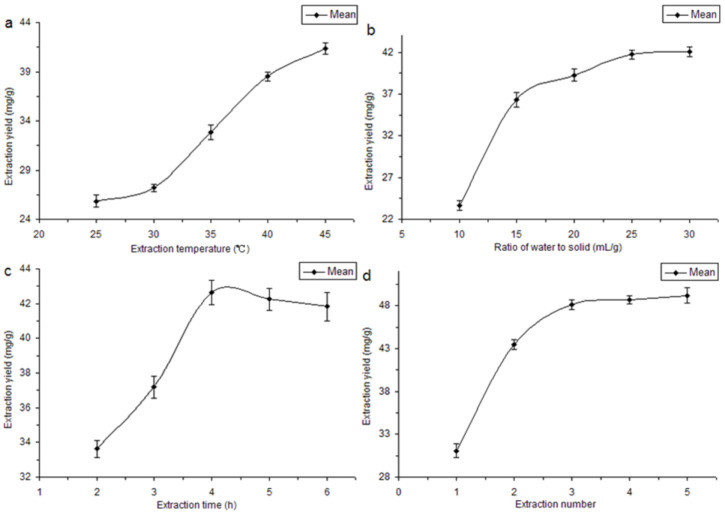
Effect of single factors on the ARPs extraction yield. (**a**) Extraction temperature, (**b**) ratio of water to solid, (**c**) extraction time, (**d**) extraction number.

A reasonable range of four factors, namely the extraction temperature, the ratio of water to solid, extraction time, and extraction cycles, affecting the extraction efficiency of ARPs are shown in Figure 1. Temperature was a key parameter for water-soluble polysaccharide extraction; however, the prepared sample often was a complex of co-extracted starch owing to the high temperature [22]. The extract of ARPs would not be tested positive in an iodine test solution reaction until the extraction temperature was below 45 °C. Therefore, 45 °C was determined as the highest extraction temperature. Then, the influence of different temperature on the yield of ARPs was measured under the constant conditions of the ratio of liquid to material 25:1 (mL/g), extraction time of 4 h, and extraction cycle 1. The extraction yield increased progressively as the temperature rose, achieving its peak at 45 °C within the evaluated temperature range (Figure 1a). Therefore, the different extraction temperature caused important effects on the yield of ARPs. Generally, as the temperature increased, the yield of extracted polysaccharides increased until its hydrolysis at a high temperature [23]. Owing to a large amount of starch in Adenophorae Radix, 40 °C was selected as the center point for further RSM experiments.

The screening of the ratio of water to solid was another important step for polysaccharide extraction [16,24]. In our investigation, we explored the impact of varying ratio of water-to-solid ratios (10:1, 15:1, 20:1, 25:1, and 30:1) on the extraction yield of A. tetraphylla. The extraction process was conducted under standardized conditions of temperature 45 °C, time 4 h, and cycle 1. As depicted in Figure 1b, the yield of ARPs reached a peak of 41.73 mg/g at a water-to-solid ratio of 25 mL/g, after which it stabilized in a dynamic equilibrium. The constituents in the raw material could not be extracted completely if the ratio of water to solid was too small. However, we observed that the yield of polysaccharides did not significantly increase at higher water-to-solid ratios. This was attributed to the diminished concentration gradient between the interior of plant cells and the external solvent, which limits the extraction efficiency [25]. Consequently, in the current study, we selected a water-to-sample ratio range of 20–30 mL/g for the extractions.

Utilizing the previously determined optimal conditions, namely an extraction temperature of 45 °C and a water-to-solid ratio of 25:1, we proceeded to examine the influence of extraction time (2, 3, 4, 5, and 6 h) on the yield of ARPs, with a single extraction cycle employed. The outcomes are graphically represented in Figure 1c. The yield of ARPs peaked at 42.64 mg/g and then exhibited a moderate decline. Shorter extraction times resulted in the incomplete dissolution of the polysaccharides. However, if the time is too long, the structure of macromolecular polysaccharides is easy to change, and the yield of polysaccharides decreases [26]. Therefore, for RSM optimization, an extraction time of 4 h was selected as the central point.

The number of extraction cycles is a critical parameter for the efficient recovery of bioactive compounds from plant materials. In our study, we systematically assessed the impact of varying extraction cycles (1, 2, 3, 4, and 5) on the yield of ARPs. Utilizing the previously established optimal conditions, five sets of samples were subjected to the extraction process. Our findings demonstrated a positive correlation between the number of extraction cycles and the ARP yield, with a plateau observed beyond three cycles, as illustrated in Figure 1d. This trend is in agreement with findings from other studies [27]. Considering the balance between extraction efficiency and resource optimization, a three-cycle extraction protocol was selected for this research.

### 2.2. Response Surface Optimization of the Extraction Conditions of ARPs

#### 2.2.1. The Model Fitting and Statistical Analysis

Table 1 presents the results of 17 designed experiments aimed at optimizing the three independent variables, X1, X2, and X3. To ensure a robust assessment, three replicate experiments (runs 13 to 17) were performed at the central point of the experimental design, enabling the estimation of the pure error sum of squares. The experimental results were analyzed by applying multiple regression analysis, and the response variable and the test variables were obtained using the following second-order polynomial equation:

Y = 57.10 + 4.94X_1_ + 2.52X_2_ + 4.38X_3_ + 0.65X_1_X_2_ − 1.89X_1_X_3_ − 0.59X_2_X_3_ + 0.83X_1_^2^ − 2.03X_2_^2^ − 1.74X_3_^2^

where X_1_, X_2_, and X_3_ were the coded values of the extraction temperature, the ratio of water to solid, and the extraction time, respectively.

The *F*-test was used to check the statistical testing of the regression equation, and the results of the ANOVA are shown in Table 2. Here, the *p*-value of the model was smaller than 0.001, and the mismatch term *p* = 0.864 > 0.5 implied that the model was suitable for predicting the yield of ARPs in this experiment. In addition, the significance of each coefficient was also measured using *F*-value and *p*-value. As can be seen in Table 2, the linear terms including all the independent variables (X_1_, X_3_), the interaction effects (X_1_ × X_3_) and two quadratic terms (X_2_^2^, X_3_^2^) had significant effects (*p* < 0.05) on the yield of ARPs. If the *F*-value was larger, the influence of parameters on the yield of ARPs was greater. Therefore, we can identify the influence of parameters on yield of ARPs in the order of extraction temperature > the ratio of water to solid > extraction time according to the *F*-value of the independent variables.

#### 2.2.2. Optimization for the Extraction Conditions of ARPs

**Figure 2:**
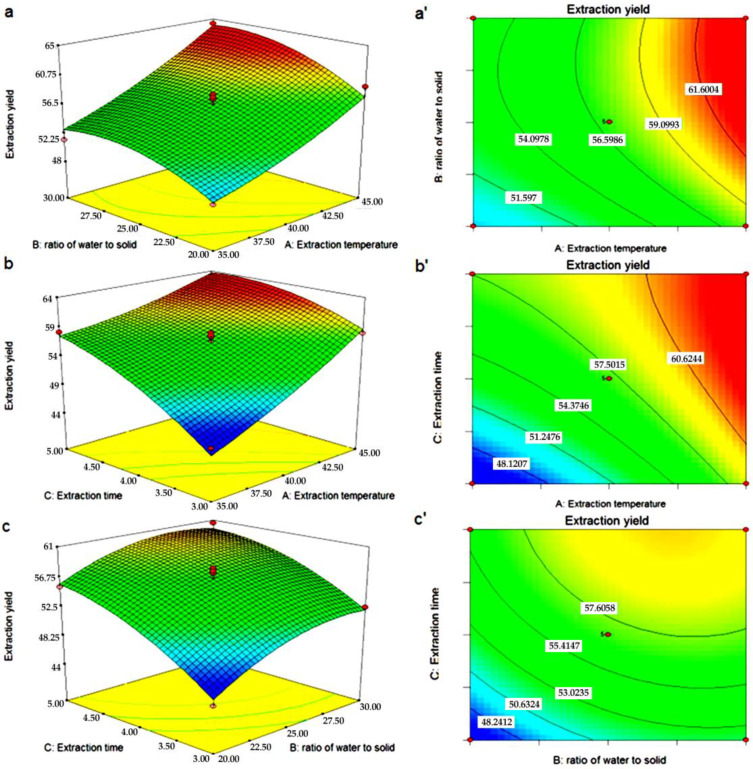
Response surface (3D) and contour plots (2D) showing the effect of different extraction parameters (X_1_: extraction temperature, °C; X_2_: the ratio of water to solid, mL/g and X_3_: extraction time, h) added on the response Y. (**a**,**a’**) Extraction temperature and ratio of water to solid, (**b**,**b’**) extraction temperature and extraction time, (**c**,**c’**) ratio of water to solid and extraction time.

The three-dimensional response surface and two-dimensional contour plots for the mutual effects of independent variables on the yield of ARPs are shown in Figure 2. These types of plots displayed the effects of two parameters on the response once, and the other factor was maintained at zero level. Figure 2a,a’ shows the mutual interactions between extraction temperature and ratio of water to solid on the yield of ARPs. It was observed that the extraction yield of ARPs increased steadily with the enhancement in the ratio of water to solid. And the trend could demonstrate that the increasing amounts of water molecules in the mixture may affect the extent of polysaccharide gelatinization [28]. From Figure 2b,b’, it can be seen that the relationship between extraction temperature and extraction time was similar to that of extraction temperature and ratio of water to solid. During the process of extraction temperature and time rising from 35 °C to 45 °C and 3 h to 5 h, respectively, an exponential increase on yield of ARPs is represented in the field. It is generally believed that the polysaccharide yield would increase significantly with the extraction conditions of higher extraction temperature and longer extraction time [29,30]. In Figure 2c,c’, the 3D surface plot and the contour plot of the interaction between ratio of water to solid and extraction time can be seen. It was observed that extraction yield of ARPs increased with the enhancement in the ratio of water to raw material. As the ratio of water to solid and the extraction time increased, the yield of ARPs also increased.

#### 2.2.3. Validation of Predictive Model

The optimum conditions for the extraction of ARPs were obtained using Design-Expert 7.0.0 software. The optimal conditions for extracting ARPs were also presented as follows: extraction temperature 45 °C, ratio of water to solid 28.47:1 mL/g, extraction time 4.60 h, and three extraction cycles, respectively. Under these parameters, the model gave a predicted ARP yield of 64.71 mg/g. To validate the adequacy of the equation model used for predicting the optimal process parameters, an optimization study was performed. Under the optimal conditions, the actual experimental yield of ARPs was 64.06 ± 0.48 mg/g (*p* > 0.05). This indicated that the model was stable and reliable for the extraction process of ARPs.

### 2.3. Promotion of HepG2 Cell Proliferation by ARPs

**Figure 3:**
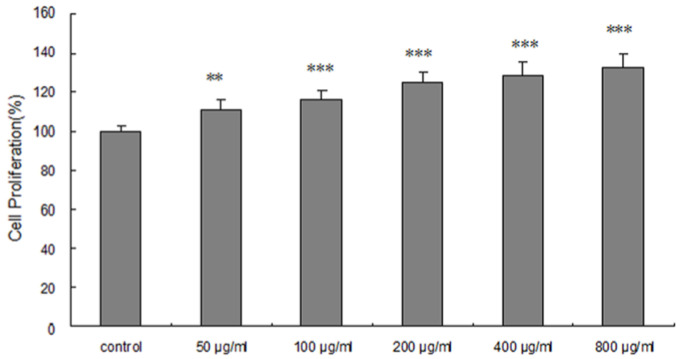
Effect of ARPs on cell proliferation in HepG2 cells. The cell proliferation was promoted by 50, 100, 200, 400, and 800 μg/mL of ARPs. Data are presented as the mean ± S.D. (n = 3). ** *p* < 0.01, *** *p* < 0.001, compared with control.

To evaluate the effect of ARPs on the growth of HepG2 cells, the cell viability was detected using the MTT assay. As shown in Figure 3, different concentrations of ARPs increased the proliferation of cells in a dose-dependent manner after 48 h incubation. Compared with the control group, the HepG2 cell proliferation was increased by 11.26% (*p* < 0.001) to 32.47% (*p* < 0.001) at a dose of 50 μg/mL to 800 μg/mL. ARPs could promote cell proliferation due to its non-toxic effect on HepG2 cells, so the glucose consumption assay would be investigated subsequently. In addition, it would be important to elucidate the mechanism by which ARPs influence cell growth and glucose metabolism in future research.

### 2.4. ARPs Promote Glucose Consumption in HepG2 Cells

Diabetes is a chronic metabolic disorder and hyperglycemia is believed to be a key characteristic. In addition, the liver has played an important role in the development of diabetes including the utilization, storage, and release of carbohydrates, fatty acid synthesis, etc. Increasing glucose uptake is widely recognized as an effective strategy for regulating glucose metabolism in diabetic patients. In our study, HepG2 cells were selected as a model due to their physiological functions, which closely resemble those of normal hepatic cells in terms of glucose and lipid metabolism [31].

**Figure 4:**
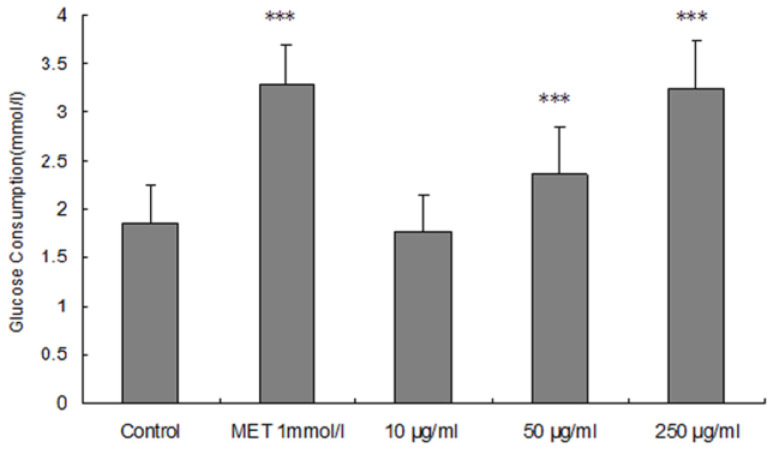
Effect of ARPs on glucose consumption in HepG2 cells. The glucose consumption was increased by 10, 50, 250 μg/mL of ARPs and 1 mM metformin. Data are presented as the mean ± S.D. (n = 3). *** *p* < 0.001, compared with control. MET, metformin.

As shown in Figure 4, there was a dose-dependent effect of ARP concentration on the glucose consumption in HepG2 cells. The glucose consumption in HepG2 cells treated with 10 μg/mL ARPs was nearly equivalent to that of the control cells. Compared with the control cells, the glucose consumption in HepG2 cells treated with 250 μg/mL ARPs increased by 75.86% (*p* < 0.001), which was slightly lower than that of the positive control metformin (77.84%, *p* < 0.001). Thus, the 250 μg/mL ARPs was almost equivalent to the glucose consumption effect of the metformin (*p* > 0.1). The significant effect of ARPs on glucose consumption in HepG2 cells suggested that ARPs might directly stimulate the glucose consumption in liver. More and more polysaccharides from medicinal plants have been found to lower glucose levels by increasing glucose consumption, and some of them might be developed as substitution therapies for diseases with insulin resistance such as type 2 diabetes mellitus [32,33].

## 3. Materials and Methods

### 3.1. Materials and Chemicals

The roots of *Adenophora tetraphylla* (Thunb.) Fisch. were collected from Shangzhi City, Heilongjiang Province, China (N 45°14′33.67″, E 127°33′54.72″), on 17 September 2021 (fruit-ripening period). All the samples were authenticated by Professor Chen Wang in the biological department, Harbin Normal University, China. A voucher specimen was deposited at the Pharmaceutical College, Heilongjiang University of Chinese Medicine, China. After removing fibrous roots, the material was dried at 50 °C for 12 h and cut into slices, then pulverized into powder, and passed through a 60-mesh sieve. The powder was kept in sealed polyethylene bags at 4 °C until use. All used solvents and chemicals were of analytical grade.

### 3.2. Extraction of ARPs

The obtained samples of fine powder (3 g) were extracted by water solvent in a designed extraction temperature, ratio of water to solid, extraction time, and number. The supernatant was obtained from the extraction solution via centrifugation at 4000 rpm for 10 min. Subsequently, ethanol was added to the supernatant to achieve a final concentration of 80% for precipitation. The precipitate was collected and then washed sequentially with anhydrous ethanol, acetone, and ether, each for three repetitions. The solid residue was reconstituted in water, and the extraction of ARPs was facilitated through a freeze-drying process, yielding a preparation that was subsequently subjected to quantitative analysis.

### 3.3. Determination of ARPs

Weigh out 5.04 mg of ARPs with precision and transfer it into a 25 mL volumetric flask. Add distilled water to dissolve the ARPs, adjust the volume to the mark, and mix thoroughly to prepare the test solution. The content of ARPs was analyzed using the phenol–sulfuric method, as previously described [34]. Pipette 1.0 mL of this solution into a 10 mL capped test tube and dilute it with distilled water up to 2 mL. Then, add 1.0 mL of a 5% phenol solution followed by 5.0 mL of concentrated sulfuric acid. Immediately place the test tube in an ice bath to cool, allow it to equilibrate to room temperature, and mix the contents well. Subsequently, immerse the test tube in a water bath at 90 °C for 20 min and allow it to cool down to room temperature once more. Measure the absorbance of the reaction mixture at 490 nm using UV spectrophotometry, and from this, calculate the content of D-anhydrous glucose equivalent in the ARP sample (m_0_). The extraction yield of the ARPs was determined using the following equation:

ARP yield (%) = m_0_/m_1_ × 100%

m_0_ (g) is the mass of ARPs; m_1_ (g) is the mass of dry powder.

### 3.4. Experimental Design and Statistical Analysis

The extraction temperature, ratio of water to solid, extraction time, and extraction number can affect the extraction efficiency of ARPs, so these four factors were screened by single-factor experiment. The factors and their levels were set as follows: the extraction temperature (25 °C, 30 °C, 35 °C, 40 °C, 45 °C), ratio of water to solid (1:15, 1:20, 1:25, 1:30, 1:35), extraction cycles (1 time, 2 times, 3 times, 4 times, 5 times), and extraction time (1 h, 2 h, 3 h, 4 h, 5 h).

Based on the outcomes from preliminary single-factor experiments, the optimal extraction parameters for ARPs were established through the BBD methodology, which incorporated three independent variables: X_1_ for extraction temperature, X_2_ for the ratio of water to solid, and X_3_ for the extraction time. Extraction cycles were fixed to 2 times, and the experimental design was carried out using BBD (Design Expert software, Trial Version 7.0.0, Stat-Ease Inc., Minneapolis, MN, USA) to determine the most influential variable for the yields of ARPs. The independent variables and levels are shown in Table 3. Referring to the experimental data of ARP content, a second-order polynomial model was adeptly fitted in accordance with the experimental design. This model serves to elucidate the correlation between the independent variables and the ARP yield, thereby facilitating the prediction of the most favorable extraction conditions.

### 3.5. Cell Culture

Human hepatoblastoma HepG2 cells were procured from the Center for Experimental Animals at Sun Yat-sen University in Guangzhou, China. The cells were cultured in Roswell Park Memorial Institute 1640 (RPMI-1640) medium, enriched with 10% fetal bovine serum, 100 U/mL penicillin, 100 µg/mL streptomycin, and 2 mM L-glutamine. The cultures were maintained in a humidified incubator with an atmosphere containing 5% CO_2_ at a temperature of 37 °C. The growth medium was replaced every two days and the cells were subcultured at 3- to 4-day intervals.

### 3.6. The Effects of ARPs on HepG2 Cell Proliferation

The effect of ARPs on HepG2 cell viability was detected using the MTT assay [35]. Cells (5 × 10^3^ cells per well) were cultured in 96-well plates for 24 h. The ARP samples were prepared using optimized extraction conditions derived from the BBD methodology. In the test groups, the cells were treated with different final concentrations of ARPs (50, 100, 200, 400, 800 μg/mL), and a control group of an equal amount of DMSO was set up. After 48 h of incubation, 100 μL of MTT reagent was added to each well. Following a 4 h incubation period, the culture medium was aspirated from each well, and 150 μL of DMSO was added. The resultant absorbance at 490 nm was quantified using a microplate reader (Thermo Multiskan MK3; Waltham, MA, USA). The cell proliferation rate was then determined by comparing the absorbance values of the experimental groups with those of the control group, which was treated solely with the vehicle.

### 3.7. Glucose Consumption Assay

The glucose consumption activity of ARPs was evaluated based on the reported method with minor modifications [36]. HepG2 cells (5 × 10^3^ cells per well) were incubated for 12 h, then the medium was supplemented with 1 mmoL/L metformin. Different final concentrations of ARPs (10, 50, 250 μg/mL) or DMSO were added to each well including the blank wells. After 24 h treatment, the glucose concentration in the mixed solutions were detected by the glucose oxidase method. The glucose consumption was calculated using the following formula:

Glucose consumption = glucose concentrations of blank wells − glucose concentrations in plated wells.

And the glucose consumption values were adjusted by the MTT assay.

### 3.8. Statistical Analysis

All experiments were carried out in triplicate, and data were expressed as mean ± standard deviation (SD). Data analysis of the single-factor experimental data was performed using SPSS 22.0 (SPSS Inc., Chicago, IL, USA). The experimental design and the determination of optimized conditions were conducted utilizing Design-Expert software. The significance of the regression coefficient was evaluated through the *F*-test and the associated *p*-value. Furthermore, the model’s adequacy was ascertained by examining the lack of fit, the coefficient of determination (*R*^2^), and the *F*-test value derived from the analysis of variance (ANOVA). A *p* value of <0.05 was considered as statistically significant.

## 4. Conclusions

In this study, we optimized the extraction parameters for ARPs using the BBD, yielding a quadratic polynomial model derived from the RSM. This model effectively quantified the impact of three critical variables: extraction temperature, ratio of water to solid, and extraction time. The refined extraction process presents a sustainable and industry-relevant alternative, characterized by streamlined operations and enhanced yields of the desired extracts. The in vitro effects of ARPs on HepG2 cell proliferation and glucose consumption were evaluated, which showed that ARPs could promote the cell proliferation and increase glucose consumption at different doses. ARPs might directly stimulate the glucose consumption in liver and showed no cytotoxicity; therefore, ARPs could probably be taken as potential natural source of antidiabetic materials. Further studies are in progress to clarify the constituents of ARPs and to elucidate the mechanism of the antidiabetic effects of ARPs.

## Figures and Tables

**Table 1:** The Box–Behnken experimental design with three independent variables.

No.	X_1_/Extraction Temperature (°C)	X_2_/Ratio of Water to Solid (mL/g)	X_3_/Extraction Time (h)	Y/Extraction Yield (mg/g)
1	40	30	3	52.42
2	35	25	5	58.16
3	45	30	4	64.28
4	35	30	4	51.32
5	45	25	3	58.01
6	45	20	4	59.18
7	40	30	5	60.52
8	35	20	4	48.82
9	40	20	5	55.42
10	35	25	3	46.15
11	45	25	5	62.46
12	40	20	3	44.97
13	40	25	4	56.45
14	40	25	4	57.62
15	40	25	4	56.02
16	40	25	4	58.09
17	40	25	4	57.31

**Table 2:** ANOVA for response surface quadratic model analysis of variance table.

Source	Sum of Squares	Degree of Freedom	Mean Square	*F*-Value	*p*-Value (Prob > F)
Model	449.49	9	49.94	27.10	0.0001
X_1_	194.83	1	194.83	105.72	<0.0001
X_2_	50.75	1	50.75	27.54	0.0012
X_3_	153.21	1	153.21	83.14	<0.0001
X_1_X_2_	1.69	1	1.69	0.92	0.3702
X_1_X_3_	14.29	1	14.29	7.75	0.0271
X_2_X_3_	1.38	1	1.38	0.75	0.4154
X_1_^2^	2.92	1	2.92	1.58	0.2487
X_2_^2^	17.36	1	17.36	9.42	0.0181
X_3_^2^	12.68	1	12.68	6.88	0.0343
Residual	12.90	7	1.84		
Lack of fit	10.02	3	3.34	4.63	0.0864
Pure error	2.88	4	0.72		
Cor total	462.39	16			
R^2^	0.9721				
Adj. R^2^	0.9362				
Pred. R^2^	0.6436				
Adequate precision	18.258				

**Table 3:** Variables and experimental design levels for response surface methodology.

Independent Variables	Coded Symbols	Levels		
		−1	0	1
Extraction temperature (°C)	X_1_	35	40	45
Ratio of water to solid (mL/g)	X_2_	20	25	30
Extraction time (h)	X_3_	3	4	5

## Data Availability

The original contributions presented in the study are included in the article, further inquiries can be directed to the corresponding author.
